# Evaluation of risk factors and assessment models for predicting venous thromboembolism in lung cancer patients

**DOI:** 10.1007/s12032-018-1120-9

**Published:** 2018-04-03

**Authors:** Joanna Rupa-Matysek, Marta Lembicz, Eliza Katarzyna Rogowska, Lidia Gil, Mieczysław Komarnicki, Halina Batura-Gabryel

**Affiliations:** 1 Department of Hematology and Bone Marrow Transplantation, Poznan University of Medical Sciences, Szamarzewskiego 84, 60-569 Poznan, Poland; 2 Department of Pulmonology, Allergology and Respiratory Oncology, Poznan University of Medical Sciences, Poznan, Poland; 3 Students Scientific Society, Poznan University of Medical Sciences, Poznan, Poland

**Keywords:** Lung cancer, Venous thromboembolism, Venous thromboembolism risk assessment models, COMPASS-CAT model

## Abstract

The aim of the study was to investigate the prognostic significance of selected risk assessment models (RAMs) for predicting venous thromboembolism (VTE) events in patients undergoing outpatient chemotherapy for lung cancer. We evaluated the following VTE-risk assessment tools: Khorana risk score (KRS), PROTECHT score, CONKO score and COMPASS-cancer-associated thrombosis score (COMPASS-CAT). Retrospective analyses were performed on 118 patients with lung cancer, 20 of whom developed VTE with a median of 2.5 months from diagnosis. Patients receiving gemcitabine-based regimen (25%), patients with a history of atrial fibrillation (AF) and patients with chronic kidney disease developed VTE more often than other patients. In the multivariate analysis, high COMPASS-CAT score (OR 8.73; 95% CI 1.01–75.22, *P *= 0.049), gemcitabine chemotherapy (OR 3.37; 95% CI 1.09–10.39, *P *= 0.035) and AF (OR 7.19; 95% CI 1.89–27.33, *P *= 0.004) were all significantly associated with VTE development. VTE occurred in; 13% (*n* = 2) of the KRS high-risk group, 17.7% (*n* = 11) of the PROTECHT high-risk group, 15% (*n* = 4) of the CONKO high-risk group and 23.8% (*n* = 20) of the COMPASS-CAT high-risk group (*n* = 84). Only the COMPASS-CAT score was able to identify 100% of patients who developed VTE, and best discriminated between patients with high and low risk of VTE development (C statistic 0.89). The ROC analysis indicated a cutoff value of 11 points (95% CI 0.821–0.962) for COMPASS-CAT for VTE development in patients with lung cancer. In conclusion, in our study of all the VTE–RAMs analyzed, the COMPASS-CAT model was the most accurate predictor of VTE development in patients with lung cancer.

## Introduction

Lung cancer is currently the most common type of malignant tumor worldwide and the leading cause of death from malignancies [1]. The incidence of venous thromboembolism (VTE) among ambulatory patients treated for lung cancer can reach 14% [2]. In addition, chemotherapeutic agents routinely administered for lung cancer therapy also influence the risk of VTE development [3, 4]. The benefits of routine primary thromboprophylaxis in ambulatory patients receiving chemotherapy for lung cancer have not been clearly demonstrated, and it is currently not recommended [5–8]. However, attempts are being made to identify patients at a high risk of VTE development, who could benefit from primary pharmacological thromboprophylaxis. Recently, a number of risk assessment models (RAMs) predicting the risk of VTE development in patients with solid tumors have been proposed, but their clinical effectiveness in particular tumors remains a matter of debate.

The best-validated model is the Khorana risk score (KRS), in which lung cancer is considered to constitute a high risk of VTE development the KRS [9]. Other RAMs, the PROTECHT score (PROphylaxis of ThromboEmbolism during CHemoTherapy) and the CONKO score are modifications of the KRS and have not been validated in a homogenous group of patients with lung cancer [10, 11]. The recently developed COMPASS-cancer-associated thrombosis (COMPASS-CAT) model seems to be most predictive of the risk of VTE development because it includes patient- and cancer-related risk factors, and comorbidities as well as the oncological treatment administered [12]. Recent studies generally undermine the effectiveness of the KRS in the prediction of VTE in lung cancer [13, 14]. Moreover, there have been no external validation studies to evaluate the COMPASS-CAT model in clinical practice. To our knowledge, the clinical usefulness of the aforementioned VTE–RAMs has not been directly compared in a group of patients with lung cancer. Therefore, the aim of our study was to validate the KRS, CONKO, PROTECHT and COMPASS-CAT models in a homogenous group of patients treated for lung cancer.

## Patients and methods

Retrospective analysis was performed on newly diagnosed patients with lung cancer who were receiving treatment at the outpatient clinic of the Department of Pulmonology, Allergology and Pulmonary Oncology at Poznan University of Medical Sciences between January 2016 and December 2016. The observation time was defined by the study end date (December 2017), disease progression and occurrence of VTE or death. The data on all-cause mortality between the first of January 2016 and the 31st of December 2017 were based on the reports of the Polish National Health Fund. Patients’ data, including complete blood counts, common comorbidities and VTE episodes, were derived from medical records available from the patient’s files. Demographic data and clinical cancer details including histopathological diagnosis, stage of disease according to the 7th edition of TNM classification and applied treatment (chemotherapy, radiotherapy, surgery and implementation of central venous catheter) were all analyzed [15]. Stages IIIB and IV according to TNM (7th edition) were considered advanced disease [15]. Different chemotherapy schedules were applied, but for our analyses platinum- or gemcitabine-based regimens were taken. None of the patients received anthracyclines-based chemotherapy. Erythropoiesis-stimulating agents were not administered.

Data on the incidence of common comorbidities including coronary artery disease, heart failure, hyperlipidemia, hypertension, atrial fibrillation (paroxysmal/persistent or permanent), stroke, diabetes, chronic obstructive pulmonary disease (COPD), asthma, chronic kidney disease and obesity were obtained. All comorbidities were diagnosed according to the most current guidelines of the relevant international societies and the appropriate treatment recorded in the documentation confirmed the diagnosis of specific diseases. The Charlson comorbidity index (CCI) and simplified comorbidity score (SCS) were also evaluated [16, 17]. The data on the use of prophylactic aspirin, clopidogrel, vitamin K antagonists (VKA) and DOACs (direct oral anticoagulants) were also collected. Additionally, any history of other malignancies, personal history of VTE and recent hospitalizations for acute medical illness within 3 months before starting treatment were also analyzed.

No routine screening for VTE was performed. The occurrence of VTE was defined as an episode of symptomatic pulmonary embolism (PE) and deep vein thrombosis (DVT) which appeared after the diagnosis of lung cancer and was confirmed by imaging tests (computed tomography angiography to detect PE or Color and Doppler ultrasound to diagnose DVT). No primary prophylaxis of VTE was administered except for the patients with atrial fibrillation (persistent/recurrent or permanent AF, *n* = 13) including low molecular weight heparin (LMWH, *n* = 8), vitamin K antagonists (VKA, *n* = 4) and 1 patient—rivaroxaban. Among the patients with AF, 83% had a CHA2DS2-VASc risk stratification score ≥ 2 and one patient had a HAS-BLED bleeding risk score above 3 [18, 19].

For the VTE- risk assessment models estimation, we classified and evaluated the studied lung cancer patients into different risk groups of VTE development according to the following VTE-risk assessment tools: the KRS, the PROTECHT score, the CONKO score and the COMPASS-CAT score [9–12]. According to the KRS, patients were categorized into high-risk (≥ 3 points) group based on the site of cancer (1 point for lung cancer), pre-chemotherapy platelet count over 350 × 10^9^/L, leukocyte count over 11 × 10^9^/L, hemoglobin below 10 g/dl and/or use of erythropoiesis-stimulating agents and a body mass index above 35 kg/m^2^ (BMI, 1 point each) [9]. The PROTECHT score is the KRS modified by adding platinum or gemcitabine-based chemotherapy (1 point each) to the predictive variables in the KRS, and a score of ≥ 3 points indicates the high-risk group for the development of VTE [10]. The CONKO score is also a modified KRS in which the BMI is replaced by the Eastern Cooperative Oncology Group (ECOG)/World Health Organization (WHO) performance status ≥ 2 (1 point), and patients with ≥ 3 points are considered as high risk for the development of VTE [11]. Another model implemented was the COMPASS-CAT model which includes cancer-related risk factors such as anthracycline treatment (6 points), time since cancer diagnosis ≤ 6 months (4 points), central venous catheter (3 points) and advanced stage of cancer (2 points); predisposing risk factors including cardiovascular risk factors (composed of at least two of the following predictors: personal history of peripheral artery disease, ischemic stroke, coronary artery disease, hypertension, hyperlipidemia, diabetes, obesity—5 points), recent hospitalization for acute medical illness (5 points) and personal history of VTE (1 point); biomarkers consisted of platelets count ≥ 350 × 10^9^/L (2 points) [12]. In the COMPASS-CAT model, high risk for VTE development is assigned to a score of 7 or more points. For the KRS and COMPASS-CAT models, a pre-chemotherapy full blood count was performed by standard methods.

Because our study involved retrospective analysis of existing data with no patient intervention or interaction, and the patient data were de-identified, the Bioethics Committee of Poznan University of Medical Sciences determined that this study was not a medical experiment and was exempt from the Bioethics Committee of Poznan University of Medical Sciences review (No 210/18). Therefore, no consent for participation was required for this study.

### Statistical analysis

Based on data from literature [20, 21], a VTE event rate of about 14% was assumed and it was calculated that at least 115 patients would be required to determine the role of RAMs with a power of 90% using a two-side test at an alpha level of 0.05 when the population size is less than 10,000.

Descriptive statistics, such as the frequency (*n*), arithmetic mean ($\bar{x}$) and standard deviation (SD), are presented for normally distributed variables. Otherwise, medians and the standard errors (SE) with interquartile ranges (25 and 75 percentiles) were used. The Shapiro–Wilk test was performed to assess normality. To compare differences between the groups, the Chi-square test was used for categorical variables and the Mann–Whitney *U* test was used for continuous variables.

Receiver operating characteristic (ROC) curve analysis was performed to determine the cutoff values for the VTE-risk assessment models (RAMs) predictive level of VTE development and for the evaluation of the VTE–RAM.s For all VTE–RAMs, we calculated the sensitivity (probability of high risk in those patients experiencing VTE), specificity (probability of high risk in those not experiencing VTE), positive predictive value (PPV, probability of high risk in those patients identified to be at high risk) and negative predictive value (NPV, probability of no VTE in those patients identified to be at low risk) for VTE development.

Univariate logistic regression was used to evaluate potential risk factors that may influence VTE. A multivariate analysis was performed with selected variables that were significant in the univariate analysis (*P *< 0.01). In each model, the odds ratio (OR) for each independent variable was determined with a confidence interval (CI) of 95%.

The probabilities of survival were estimated via the Kaplan–Meier method, and univariate comparisons were performed via the log-rank test. A *P* value < 0.05 was regarded as statistically significant. The statistical analyses were performed with STATISTICA 13 and STATISTICA Medical Package (StatSoft, Inc., Tulsa, Oklahoma, USA).

## Results

A total of 118 adult patients with newly diagnosed lung cancer including non-small-cell carcinoma (NSCLC, *n* = 97) or small-cell carcinoma (SCLC, *n* = 21) undergoing chemotherapy were enrolled in the study. All patients were Caucasian, with a median age of 64 years (range 39–83 years), and 58% were male. The median observation time was 14 months (range 1–24). Detailed data concerning the baseline characteristics of the studied population and comparisons of the patients with or without VTE are shown in Table 1.

**Table 1:** Baseline characteristics of the studied population and comparisons of the patients with or without VTE

Characteristics	*n* (%)	VTE^a^ *n* = 20	Non-VTE^a^ *n* = 98	*P* value
*Demographic data*				
Median age, range years	64 (39–83)	65 (39–79)	63 (46–83)	0.3993
Gender, male *n* (%)	68 (58%)	12 (60%)	56 (57%)	0.8137
WHO performance status ≥ 2	18 (15%)	4 (20%)	14 (14%)	0.5172
BMI ≥ 35 kg/m^2^	6 (0.05%)	1 (5%)	5 (5%)	0.9849
*Comorbidity variables*				
Coronary artery disease	32 (27%)	8 (40%)	24 (24%)	0.1551
Heart failure	5 (0.04%)	0 (0%)	5 (5%)	0.3019
Hyperlipidemia	54 (46%)	10 (50%)	44 (45%)	0.6764
Hypertension	71 (60%)	9 (45%)	62 (63%)	0.1284
Atrial fibrillation	13 (11%)	7 (35%)	6 (6%)	0.0002
Stroke	2 (0.02%)	1 (5%)	1 (15)	0.2089
Diabetes	24 (20%)	5 (25%)	19 (19%)	0.5699
COPD	25 (21%)	5 (25%)	20 (20%)	0.6469
Asthma	6 (0.05%)	1 (5%)	5 (5%)	0.9849
Chronic kidney disease	15 (13%)	6 (30%)	9 (9%)	0.0109
Obesity (BMI ≥ 35 kg/m^2^)	6 (0.05%)	1 (5%)	5 (5%)	0.9849
Presence of comorbidities	102 (86%)	17 (85%)	85 (87%)	0.8364
High CCI score (≥ 3 points)	47 (40%)	9 (45%)	38 (39%)	0.6043
High SCS score (score > 9)	30 (25%)	7 (35%)	23 (23%)	0.2805
History of other malignancies	24 (20%)	3 (15%)	21 (21%)	0.5151
Personal history of VTE	5 (0.04%)	2 (10%)	3 (3%)	0.1604
Recent hospitalization for acute medical illness	66 (56%)	20 (100%)	46 (47%)	< 0.0001
Antiplatelet agents (Aspirin)	27 (23%)	5 (25%)	22 (22%)	0.8045
Anticoagulants	13 (11%)	7 (35%)	6 (6%)	0.0017
*Histological type*				
Squamous cell carcinoma	37 (31%)	5 (25%)	32 (33%)	0.5016
Adenocarcinoma	57 (48%)	13 (65%)	44 (45%)	0.1011
Small-cell carcinoma	21 (18%)	2 (10%)	19 (19%)	0.3171
Adenoid cystic adenoma	1 (0.01%)	0 (0%)	1 (1%)	–
NOS lung carcinoma	2 (0.02%)	0 (0%)	2 (2%)	–
*Lung cancer-stage according TNM 7th edition*				
Stage Ia	1 (0.01%)	0 (0%)	1 (1%)	–
Stage Ib	4 (0.03%)	0 (0%)	4 (4.1%)	–
Stage IIa	4 (0.03%)	0 (0%)	4 (4.1%)	–
Stage IIb	6 (0.05%)	1 (5%)	5 (5.2%)	–
Stage IIIa	16 (14%)	0 (0%)	16 (16.3%)	–
Stage IIIb	15 (13%)	3 (15%)	12 (12.2%)	0.7356
Stage IV	72 (61%)	16 (80%)	56 (57.1%)	0.0561
Advanced disease^b^	76 (64%)	14 (70%)	62 (63%)	0.5665
*Treatment variables*				
Surgery	5 (4%)	0 (0%)	5 (5%)	–
Radiation	6 (5%)	1 (5%)	5 (5%)	–
Chemotherapy alone	39 (33%)	8 (40%)	31 (32%)	0.4683
Complex treatment (chemotherapy with surgery or radiation)	68 (58%)	11 (55%)	57 (58%)	0.7944
Gemcitabine-based chemotherapy	25 (21%)	9 (45%)	16 (16%)	0.0042
Platinum-based chemotherapy	96 (81%)	14 (70%)	82 (84%)	0.1525
Central venous catheter	7 (6%)	1 (5%)	6 (6%)	0.8465
*Pre-chemotherapy CBC variables*				
Platelet count > 350 × 10^9^/l	40 (34%)	6 (30%)	34 (35%)	0.6861
Leukocyte count > 11 × 10^9^/l	26 (20%)	4 (20%)	22 (22%)	0.8097
Hemoglobin < 10 g/dl × 10^9^/l	4 (10%)	2 (10%)	2 (2%)	0.0731
*Outcome variables*				
Death	76 (64%)	16 (80%)	60 (61%)	0.1100

^a^The percentages are related to the numbers presented in the first column of the same line

^b^Advanced disease: stage ≥ III B according to the 7th edition of TNM classification

*CBC* complete blood count; *CCS* Charlson comorbidity index; *SCS* simplified comorbidity Score

*P* < 0.05—statistically significant

Overall, 20 (16.9%) patients developed venous thromboembolism in the median 2.5 months (25th–75th percentile: 1–7.5), of whom 7 were presented with symptomatic pulmonary embolism, 7 cases were with deep vein thrombosis of lower extremities and 6 patients had both pulmonary embolism and deep vein thrombosis of lower extremities. No impact of the central venous catheter implementation on VTE development was found (1 vs. 5, *p* = 0.8465). Patients with a history of paroxysmal/persistent or permanent AF developed VTE more often than other patients (35 vs. 6%, *P *= 0.0002). Despite the fact that all patients with AF received anticoagulation or thromboprophylaxis with LMWH, 7 out of 13 patients developed VTE including 5 patients on LMWH, 1 patient on VKA and 1 case on rivaroxaban. In comparison with the patients without AF, those with AF less often had heart failure (2 vs. 11, *P *= 0.034). There were no significant differences in other analyzed variables between the patients with or without AF. Comparison of patients with or without VTE demonstrated that patients with chronic kidney disease (CKD) had VTE events more often than patients without CKD (30 vs. 9%, *P *= 0.0109). Furthermore, patients with VTE had more often been hospitalized for acute medical illness within 3 months before starting chemotherapy than the patients without VTE (100 vs. 47%, *P* < 0.0001). In our study, 80% of patients received platinum-based chemotherapy and 21% of patients had gemcitabine-based regimen. None of the patients received both gemcitabine and platinum at the same time. Furthermore, patients receiving the gemcitabine-based regimen (25%) had a higher incidence of VTE than the patients undergoing other treatment (45 vs. 16%, *P *= 0.0042). No further differences were found between patients with or without gemcitabine-based chemotherapy. There were no significant differences in other patient- and cancer-related risk factors, presence of comorbidities or studied comorbidity scores between the VTE and non-VTE group (Table 1).

VTE occurred in 13% (*n* = 2) of the high-risk group (*n* = 15) and in 17.5% (*n* = 18) of the intermediate group (*n* = 103) according to the KRS, in 17.7% (*n* = 11) of the high-risk group (*n* = 62) and in 16% (*n* = 9) of the low group (*n* = 56) according to the PROTECHT, in 15% (*n* = 4) of the high-risk group (*n* = 26) and in 17.4% (*n* = 16) of the low group (*n* = 92) according to the CONKO and in 23.8% (*n* = 20) of the high-risk group (*n* = 84) and in 0% of the low group (*n* = 34) according to the COMPASS-CAT VTE–RAM. Only the COMPASS-CAT score was able to identify 100% of patients who developed VTE out of the high-risk group (*n* = 84). Detailed comparisons of the patients with or without VTE according to risk assessment models for VTE development are provided in Table 2.

**Table 2:** Comparison of the characteristics of patients with and without VTE according to risk assessment models for VTE development

	Overall population *n* = 118	VTE group during follow-up^a^ *n* = 20	Non-VTE group during follow-up^a^ *n* = 98
High KRS^b^	15 (13%)	2 (10%)	13 (13%)
High PROTECHT^c^	62 (52%)	11 (55%)	51 (52%)
High CONKO^d^	26 (22%)	4 (20%)	22 (22%)
High COMPASS^e^	84 (71%)	20 (100%)	64 (65%)

^a^The percentages are related to the numbers presented in the first column of the same line

^b^According to the Khorana risk score (KRS) for VTE-risk assessment model (VTE–RAM)

^c^According to the PROTECHT score for VTE–RAM

^d^According to the CONKO score for VTE–RAM

^e^According to the COMPASS-CAT score for VTE-RAM

*P *< 0.05: statistically significant

**Fig. 1:**
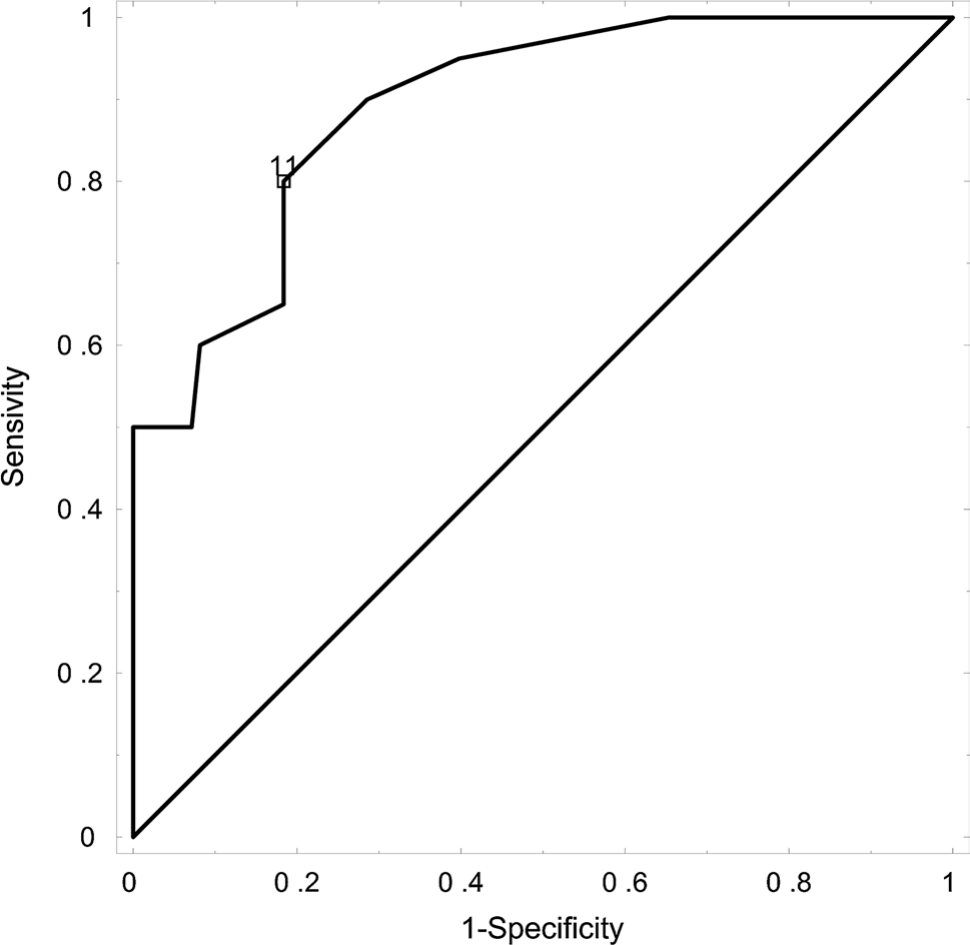
Receiver operating characteristic (ROC) curve analysis of COMPASS-CAT RAM for the prediction of VTE development in lung cancer patients

For a high KRS score, the sensitivity was 10%, the specificity 100%, the PPV 17% and the NPV 83%. For a high PROTECH score, the sensitivity was 20%, the specificity 78%, the PPV 18% and the NPV 84%. For a high CONKO score, the sensitivity was 55%, the specificity 48%, the PPV 15% and the NPV 82%. For a high COMPASS-CAT score, the sensitivity was 100%, the specificity 35%, the PPV 24% and the NPV 100%. In our patients treated for lung cancer, the COMPASS-CAT score best discriminated between patients with high and low risk of VTE development (C statistic 0.89). VTE rates and negative and positive predictive values for the development of VTE based on the VTE-RAMs are presented in Table 3. The ROC analysis indicated a cutoff value of 11 points (95% CI 0.821–0.962) for COMPASS-CAT RAM with 95% sensitivity and 61% specificity (ROC AUC = 0.891, SE = 0.036) for the development of VTE, Fig. 1.

**Table 3:** VTE rates and negative and positive predictive values for the development of VTE based on the VTE-risk assessment models in lung cancer patients

Risk group	Patients, *n*	VTE, *n*	PPV, %	NPV, %	Sensitivity, %	Specificity, %	C statistic
*KRS score*							
Low/intermediate	103	18	13	0	100	87	0.81
High	15	2	17	83	10	100	
*PROTECHT score*							
Low/intermediate	56	9	17	0	100	0	0.51
High	62	11	18	84	55	48	
*CONKO score*							
*Low/intermediate*	92	16	17	0	100	0	0.49
*High*	26	4	15	82	20	78	
*COMPASS-CAT score*							
Low	364	53	17	0	100	0	0.89
High	64	11	24	100	100	35	

*PPV* positive predictive value; *NPP* negative predictive value; *VTE* venous thromboembolism

### Factors associated with VTE and overall survival

A high COMPASS-CAT score, gemcitabine chemotherapy, atrial fibrillation, recent hospitalization for acute medical illness and chronic kidney disease were significantly associated with an increased risk of VTE based on the univariate analysis (Table 4). In univariate analysis, there was a trend toward an increased risk of VTE in patients with advanced disease and a pre-chemotherapy hemoglobin value < 10 g/dl. When variables were included in the multivariate analysis, high COMPASS-CAT score (OR 8.73; 95% CI 1.01–75.22, *P *= 0.049), gemcitabine chemotherapy (OR 3.37; 95% CI 1.09–10.39, *P *= 0.035) and atrial fibrillation (OR 7.19; 95% CI 1.89–27.33, *P *= 0.004) remained significant for VTE development (Table 5).

**Table 4:** Univariate analyses of determining factors that affect VTE development in patients with lung cancer

Factor	Odds ratio (95% CI)	*P*
High COMPASS-CAT score	9.65 (1.24–75.24)	0.031
Gemcitabine chemotherapy	4.12 (1.09–10.39)	0.006
Atrial fibrillation	8.26 (2.40–28.41)	0.001
Recent hospitalization for acute medical illness	0.02 (0.01–0.14)	0.001
Chronic kidney disease	4.24 (1.31–13.75)	0.017
Advanced disease	0.91 (0.29–2.84)	0.868

**Table 5:** Multivariate analyses of determining factors that affect VTE development in patients with lung cancer

Factor	Odds ratio (95% CI)	*P*
High COMPASS-CAT score	8.73 (1.01–75.22)	0.049
Gemcitabine-based chemotherapy	3.37 (1.09–10.39)	0.035
Atrial fibrillation	7.19 (1.89–27.33)	0.004

During a median follow-up of 14 months (25th–75th percentile: 7–24 months), 76 patients (64%) died, including 16 patients from the group with VTE and 60 patients from the group without VTE. The median observation time in the group of patients who developed VTE was 14 months (25th–75th percentile: 8–22), in the non-VTE group it was 14 months (25th–75th percentile: 7–24). Although patients with a high Simplified comorbidity score (SCS > 9) had an increased risk of death (25 vs. 5, *P *= 0.0122), no impact of SCS and CCI on VTE development was found, Table 1.

## Discussion

Herein, we present a study comparing VTE-RAMs in a homogenous group of patients with lung cancer undergoing ambulatory chemotherapy. We demonstrated that with the use of the COMPASS-CAT score we could predict VTE in this group of patients with almost 100% accuracy. To our knowledge, despite the recent debates regarding the risk factors and methods of risk assessment for VTE in various cancers, this is the first such analysis with regard to lung cancer.

Patients with lung cancer are known to have a substantial risk of VTE development. The reported frequency of VTE in outpatient lung cancer patients varies from 5 to 14% [2, 13, 20–22]. VTE in patients with lung cancer results in increased mortality, higher treatment costs and lower quality of life [21, 23, 24]. To date, routine primary thromboprophylaxis in ambulatory patients with lung cancer has not been recommended [5–8]. Several attempts have been made to examine the effectiveness of primary thromboprophylaxis in patients with lung cancer. The FRAGMATIC study revealed a significantly lower rate of VTE in patients receiving dalteparin, but without an impact on overall prognosis and a higher incidence of bleeding [25]. Ek et al. [26] in a RASTEN trial including patients with SCLC showed similar results. Most of the studies conclude that the benefit from primary thromboprophylaxis should be weighed against the risk of bleeding [27]. In order to identify those patients at a particularly high risk of VTE who could benefit from primary thromboprophylaxis, several RAMs have been proposed, based on heterogeneous groups of patients with common tumors. Most of these RAMs have not yet been validated with a group of patients with lung cancer.

The KRS was originally derived from a cohort of outpatients with common malignancies (patients with lung cancer accounted for 20% of the group), undergoing chemotherapy, with a median observation time of 2.5 months. In the original cohort, it appeared to be predictive of VTE [9]; however, subsequent studies did not confirm the efficacy of KRS in the prediction of VTE in cancer patients [13, 28, 29]. Moreover, previous attempts to validate the KRS in patients with lung cancer did not confirm its efficacy in this particular malignancy [14], which is in line with our results. In the present study, KRS also failed in risk stratification for VTE.

The PROTECHT score is a modification of the KRS including chemotherapy agents related to high risk of VTE (platinum, gemcitabine) [10]. These agents are routinely used in treatment of advanced lung cancer and both agents increase the risk of VTE [3, 4, 30], particularly when administered together [31]. In the studied group, none of the patients received both agents in a combined chemotherapy regimen, although in some patients they were administered consecutively. However, despite the widespread use of these chemotherapeutics in the study group there was no correlation between the PROTECHT score and incidence of VTE. In line with the findings of Barni et al. [32], the presented study demonstrated an increased incidence of VTE in patients receiving gemcitabine when compared to other agents. In our study, a gemcitabine-based regimen was associated with approximately 3.0-fold increase odds for VTE occurrence.

The CONKO score, originally validated in ambulatory patients with pancreatic cancer, modifies KRS by replacing BMI with performance status (PS) [11]. Poor PS has also been found to be predictive of VTE in patients with non-small-cell lung cancer [33]. Because the studied group in our study comprised patients eligible for ambulatory chemotherapy, the PS according to ECOG classification in the majority of them was 0–1 points. Thus, patients with PS ≥ 2 were underrepresented (*n* = 18) in our study as they were assigned 1 point in the CONKO score, which resulted in the high occurrence of intermediate-risk patients in the study group. This might explain the poorer performance of this score in our study group.

The recently proposed COMPASS-CAT score includes a number of variables related to patient characteristics, comorbidities, tumors and treatment [12]. In the original cohort, lung cancer patients accounted for 13.3%. A score of 7 points indicated a high risk of VTE [12]. We found the COMPASS-CAT score proved to be the only discriminative model among the VTE–RAMs analyzed in our study. However, in contrast to the original work, in our group of patients with lung cancer we identified a score of 11 points as a cutoff point indicating a high risk of VTE development. Our result is encouraging and can improve the COMPASS-CAT performance in lung cancer patients. Furthermore, this score is applicable not only at the beginning of the oncologic treatment, but also in patients already receiving chemotherapy, allowing re-assessment of the risk of VTE during treatment.

We also attempted to identify specific patient–related risk factors for VTE, which could be considered independently of the proposed RAMs or indicate clinically important characteristics not included in the RAMs. In the univariate analysis, a recent hospitalization for acute medical illness before starting oncological treatment was a factor related to an increased risk of VTE, independent of the COMPASS-CAT score, which is consistent with previous observations [34]. However, this finding was not confirmed in multivariate analysis.

Moreover, in the present study the impact of comorbidities on the risk of VTE development was analyzed. The association between CKD and VTE occurrence has previously been demonstrated in the general population [35], but in cancer patients with CKD the data are contradictory [36, 37]. In the univariate model, the patients with lung cancer and CKD had an above 4.0-fold increased risk of VTE. As our patients were eligible for ambulatory chemotherapy and had less advanced stages of CKD, the increased risk of VTE related to CKD was not confirmed in the multivariable model which determined factors that may affect VTE development in our patients with lung cancer.

Atrial fibrilation (AF) is known as a factor related with VTE in the general population [38]. In the presented study, AF was identified in both univariate and multivariate analyses as an independent risk factor for VTE events. Despite anticoagulation therapy (vitamin K antagonists, VKA or non-VKA oral anticoagulants, NOACs) or thromboprophylaxis with LMWH, AF was associated with an approximately 7.0-fold increase in the odds for VTE development. Therefore, atrial fibrillation appears to be an especially strong risk factor of VTE and could be considered in future RAMs tailored for lung cancer.

Currently, there are no specific recommendations for chronic anticoagulation in patients with active cancer and non-valvular atrial fibrillation. As in the general population the assessment of the risk-to-benefit ratio between thrombotic risk (a CHA2DS2-VASc score greater than or equal to 2) and bleeding risk (a HAS-BLED bleeding risk score below 3) should be considered [18, 19]. VTE occurred despite anticoagulation, which illustrates a strong tendency for the development of VTE in this group of patients. Our observation is in line with previous studies on cancer patients with AF that reports an elevated risk of VTE despite prophylaxis with VKA [39] or other anticoagulants [40]. In our study, the limited number of patients with lung cancer and AF on various anticoagulants did not allow for the determination of the optimal therapy, thus making the results inconclusive in this area and require further studies necessary.

In our study the highest incidence of VTE was observed within the first months after the beginning of treatment (median 2.5 months). Recent studies also reported the highest tendency for VTE development in the first few months [21, 41]. This finding supports the need for pre-chemotherapy assessment of the risk of VTE [42].

The main strength of the present study is the novel direct comparison of RAMs designed for the prediction of VTE in a representative, homogenous group of patients with lung cancer, treated in a single reference hospital. As the patients received the majority of their treatment on this one site, the collected clinical data were complete and reflected the real incidence of symptomatic VTE in the study group. The main limitation of the study is its retrospective character. Moreover, because there was no routine screening for VTE, some cases of asymptomatic VTE may have been missed.

In conclusion, our study indicated the COMPASS-CAT score as the RAM most effective at predicting VTE in lung cancer, however, only after taking into account the modified high-risk cutoff point. These results underline the need for a RAM tailored especially for patients with lung cancer.

## References

[1] Siegel RL, Miller KD, Jemal A (2018). Cancer statistics, 2018. Cancer J Clin..

[2] Connolly GC, Dalal M, Lin J, Khorana AA (2012). Incidence and predictors of venous thromboembolism (VTE) among ambulatory patients with lung cancer. Lung Cancer.

[3] Mellema WW, van der Hoek D, Postmus PE, Smit EF (2014). Retrospective evaluation of thromboembolic events in patients with non-small cell lung cancer treated with platinum-based chemotherapy. Lung Cancer.

[4] Dasanu CA (2008). Gemcitabine: vascular toxicity and prothrombotic potential. Expert Opin Drug Saf..

[5] Lyman GH, Bohlke K, Khorana AA, Kuderer NM, Lee AY, Arcelus JI (2015). Venous thromboembolism prophylaxis and treatment in patients with cancer: American society of clinical oncology clinical practice guideline update 2014. J Clin Oncol.

[6] Mandala M, Falanga A, Roila F, Group EGW (2011). Management of venous thromboembolism (VTE) in cancer patients: ESMO clinical practice guidelines. Ann Oncol..

[7] Farge D, Bounameaux H, Brenner B, Cajfinger F, Debourdeau P, Khorana AA (2016). International clinical practice guidelines including guidance for direct oral anticoagulants in the treatment and prophylaxis of venous thromboembolism in patients with cancer. Lancet Oncol..

[8] Network. NCC. National Comprehensive Cancer Network (Version 1. 2017). https://www.nccn.org/professionals/physician_gls/pdf/meloid_growth.pdf. Accessed 10 Feb 2018.

[9] Khorana AA, Kuderer NM, Culakova E, Lyman GH, Francis CW (2008). Development and validation of a predictive model for chemotherapy-associated thrombosis. Blood.

[10] Verso M, Agnelli G, Barni S, Gasparini G, LaBianca R (2012). A modified Khorana risk assessment score for venous thromboembolism in cancer patients receiving chemotherapy: the Protecht score. Intern Emerg Med.

[11] Pelzer U, Sinn M, Stieler J, Riess H (2013). Primary pharmacological prevention of thromboembolic events in ambulatory patients with advanced pancreatic cancer treated with chemotherapy?. Dtsch Med Wochenschr.

[12] Gerotziafas GT, Taher A, Abdel-Razeq H, AboElnazar E, Spyropoulos AC, El Shemmari S (2017). A predictive score for thrombosis associated with breast, colorectal, lung, or ovarian cancer: the prospective COMPASS-cancer-associated thrombosis study. Oncologist.

[13] van Es N, Di Nisio M, Cesarman G, Kleinjan A, Otten HM, Mahe I (2017). Comparison of risk prediction scores for venous thromboembolism in cancer patients: a prospective cohort study. Haematologica.

[14] Mansfield AS, Tafur AJ, Wang CE, Kourelis TV, Wysokinska EM, Yang P (2016). Predictors of active cancer thromboembolic outcomes: validation of the Khorana score among patients with lung cancer. J Thromb Haemost.

[15] Goldstraw P, Crowley J, Chansky K, Giroux DJ, Groome PA, Rami-Porta R (2007). The IASLC lung cancer staging project: proposals for the revision of the TNM stage groupings in the forthcoming (seventh) edition of the TNM Classification of malignant tumours. J Thorac Oncol..

[16] Charlson ME, Pompei P, Ales KL, MacKenzie CR (1987). A new method of classifying prognostic comorbidity in longitudinal studies: development and validation. J Chronic Dis..

[17] Colinet B, Jacot W, Bertrand D, Lacombe S, Bozonnat MC, Daures JP (2005). A new simplified comorbidity score as a prognostic factor in non-small-cell lung cancer patients: description and comparison with the Charlson’s index. Br J Cancer.

[18] January CT, Wann LS, Alpert JS, Calkins H, Cigarroa JE, Cleveland JC (2014). 2014 AHA/ACC/HRS guideline for the management of patients with atrial fibrillation. A report of the American College of Cardiology/American Heart Association Task Force on practice guidelines and the heart rhythm society. Circulation.

[19] Kirchhof P, Benussi S, Kotecha D, Ahlsson A, Atar D, Casadei B (2017). 2016 ESC guidelines for the management of atrial fibrillation developed in collaboration With EACTS. Rev Esp Cardiol (Engl Ed)..

[20] Tagalakis V, Levi D, Agulnik JS, Cohen V, Kasymjanova G, Small D (2007). High risk of deep vein thrombosis in patients with non-small cell lung cancer: a cohort study of 493 patients. J Thorac Oncol..

[21] Kourelis TV, Wysokinska EM, Wang Y, Yang P, Mansfield AS, Tafur AJ (2014). Early venous thromboembolic events are associated with worse prognosis in patients with lung cancer. Lung Cancer.

[22] Chew HK, Wun T, Harvey D, Zhou H, White RH (2006). Incidence of venous thromboembolism and its effect on survival among patients with common cancers. Arch Intern Med.

[23] Khorana AA, Dalal MR, Lin J, Connolly GC (2013). Health care costs associated with venous thromboembolism in selected high-risk ambulatory patients with solid tumors undergoing chemotherapy in the United States. ClinicoEcononmics Outcomes Res..

[24] Khorana AA, Francis CW, Culakova E, Kuderer NM, Lyman GH (2007). Thromboembolism is a leading cause of death in cancer patients receiving outpatient chemotherapy. J Thromb Haemost.

[25] Macbeth F, Noble S, Evans J, Ahmed S, Cohen D, Hood K (2016). Randomized phase III trial of standard therapy plus low molecular weight heparin in patients with lung cancer: FRAGMATIC trial. J Clin Oncol.

[26] Ek L, Gezelius E, Bergman B, Bendahl PO, Anderson H, Sundberg J (2017). Randomized phase III trial of low molecular weight heparin enoxaparin in addition to standard treatment in small cell lung cancer: the RASTEN trial. Ann Oncol.

[27] Di Nisio M, Porreca E, Candeloro M, De Tursi M, Russi I, Rutjes AW (2016). Primary prophylaxis for venous thromboembolism in ambulatory cancer patients receiving chemotherapy. Cochrane Database Syst Rev.

[28] van Es N, Franke VF, Middeldorp S, Wilmink JW, Buller HR (2017). The Khorana score for the prediction of venous thromboembolism in patients with pancreatic cancer. Thromb Res.

[29] Agnelli G, George DJ, Kakkar AK, Fisher W, Lassen MR, Mismetti P (2012). Semuloparin for thromboprophylaxis in patients receiving chemotherapy for cancer. N Engl J Med.

[30] Khorana AA, Dalal M, Lin J, Connolly GC (2013). Incidence and predictors of venous thromboembolism (VTE) among ambulatory high-risk cancer patients undergoing chemotherapy in the United States. Cancer.

[31] Numico G, Garrone O, Dongiovanni V, Silvestris N, Colantonio I, Di Costanzo G (2005). Prospective evaluation of major vascular events in patients with nonsmall cell lung carcinoma treated with cisplatin and gemcitabine. Cancer.

[32] Barni S, Labianca R, Agnelli G, Bonizzoni E, Verso M, Mandala M (2011). Chemotherapy-associated thromboembolic risk in cancer outpatients and effect of nadroparin thromboprophylaxis: results of a retrospective analysis of the PROTECHT study. J Transl Med..

[33] Shen Q, Dong X, Tang X, Zhou J (2017). Risk factors and prognosis value of venous thromboembolism in patients with advanced non-small cell lung cancer: a case-control study. J Thorac Dis..

[34] Ashrani AA, Gullerud RE, Petterson TM, Marks RS, Bailey KR, Heit JA (2016). Risk factors for incident venous thromboembolism in active cancer patients: a population based case-control study. Thromb Res.

[35] Cheung KL, Zakai NA, Folsom AR, Kurella Tamura M, Peralta CA, Judd SE (2017). Measures of kidney disease and the risk of venous thromboembolism in the REGARDS (Reasons for Geographic and Racial Differences in Stroke) study. Am J Kidney Dis.

[36] Konigsbrugge O, Lotsch F, Zielinski C, Pabinger I, Ay C (2014). Chronic kidney disease in patients with cancer and its association with occurrence of venous thromboembolism and mortality. Thromb Res.

[37] Kooiman J, den Exter PL, Cannegieter SC, le Cessie S, del Toro J, Sahuquillo JC (2013). Impact of chronic kidney disease on the risk of clinical outcomes in patients with cancer-associated venous thromboembolism during anticoagulant treatment. J Thromb Haemost.

[38] Enga KF, Rye-Holmboe I, Hald EM, Lochen ML, Mathiesen EB, Njolstad I (2015). Atrial fibrillation and future risk of venous thromboembolism: the Tromso study. J Thromb Haemost.

[39] Ording AG, Horvath-Puho E, Adelborg K, Pedersen L, Prandoni P, Sorensen HT (2017). Thromboembolic and bleeding complications during oral anticoagulation therapy in cancer patients with atrial fibrillation: a Danish nationwide population-based cohort study. Cancer Med.

[40] Pritchard ER, Murillo JR, Putney D, Hobaugh EC (2017). Single-center, retrospective evaluation of safety and efficacy of direct oral anticoagulants versus low-molecular-weight heparin and vitamin K antagonist in patients with cancer. J Oncol Pharm Pract.

[41] Huang H, Korn JR, Mallick R, Friedman M, Nichols C, Menzin J (2012). Incidence of venous thromboembolism among chemotherapy-treated patients with lung cancer and its association with mortality: a retrospective database study. J Thromb Thrombolysis.

[42] Lyman GH, Khorana AA, Kuderer NM, Lee AY, Arcelus JI, Balaban EP (2013). Venous thromboembolism prophylaxis and treatment in patients with cancer: American society of clinical oncology clinical practice guideline update. J Clin Oncol.

